# Musculoskeletal symptoms in SARS-CoV-2 (COVID-19) patients

**DOI:** 10.1186/s13018-020-01702-w

**Published:** 2020-05-18

**Authors:** Lucio Cipollaro, Lorenzo Giordano, Johnny Padulo, Francesco Oliva, Nicola Maffulli

**Affiliations:** 1 Department of Musculoskeletal Disorders, Faculty of Medicine and Surgery, University of Salerno, Salerno, Italy; 2 Department of Medicine, Surgery and Dentistry, University of Salerno, Via S. Allende, 84081 Baronissi, SA Italy; 3 Department of Biomedical Sciences for Health, Università degli Studi di Milano, Milan, Italy; 4 Centre for Sports and Exercise Medicine, Barts and The London School of Medicine and Dentistry, Mile End Hospital, Queen Mary University of London, 275 Bancroft Road, London, E1 4DG England; 5 Institute of Science and Technology in Medicine, Keele University School of Medicine, Thornburrow Drive, Stoke on Trent, England

The novel SARS-CoV-2 (COVID-19) became a pandemic on 11 March 2020. The epidemiological picture is constantly evolving, and on 13 May, 4,170,424 cases and 287,399 confirmed deaths have been reported (WHO Report). People with COVID-19 infection may show several symptoms, including fever, cough, nausea, vomiting, dyspnea, myalgia, fatigue, arthralgia, headache, diarrhea, and rarely arthritis [1]. COVID-19 clinical features range from asymptomatic patients to acute respiratory distress syndrome (ARDS) and multiple organ dysfunction [2, 3]. Influenza symptoms are associated with a cascade of inflammatory mediators. Interleukin-6 (IL-6) and tumor necrosis factor-α (TNF- α) levels in plasma and upper respiratory secretions directly correlate with the magnitude of viral replication, fever, and respiratory and systemic symptoms, including musculoskeletal clinical manifestations [4, 5] Musculoskeletal symptoms such as fatigue, myalgia and arthralgia are common COVID-19 symptoms, but their prevalence has not yet been systematically investigated [6, 7]. We collected the published clinical data of the past 5 months to ascertain the prevalence of musculoskeletal symptoms and epidemiological characteristics published worldwide in COVID-19 patients.

Data were tabulated using Microsoft Excel^TM^ 2020 V.16.34. The value was showed as mean ± SD. Student *t* test was used to reveal musculoskeletal symptoms between the total sample. To assess the incidence for each clinical variable, frequency analysis was performed. Regression analysis (*R*^*2*^) was used to examine correlations between the total sample and musculoskeletal symptoms extracted. The level of significant was set at *p* < 0.05.

The relevant reference and the data collected from the included articles are indicated in Tables 1 and 2.

**Table 1:** Demographics

Study (year)	No. of patients	Sex	Age (mean SD or median IQR)	Study design	Country
Zheng et al. [8]	99	M 51 F 48	49.40 (SD 18.45)	Retrospective single center	China
Lei et al. [9]	34	M 14 F 20	55 (43–63)	Retrospective single center	China
Mo et al. [10]	155	M 86 F 69	54 (42–66)	Retrospective single center	China
Qian et al. [11]	91	M 37 F 54	50 (54–80)	Retrospective multi-center	China
Ma et al. [12]	37	M 20 F 17	62 (59–70)	Retrospective single center	China
Jin et al. [13]	651	M 331 F 246	46.0 (32–60)	Retrospective multi-center	China
Zheng et al. [14]	161	M 80 F 81	45.0 (33.5–57)	Retrospective single center	China
Wang et al. [15]	80	M 31 F 49	39.0 (32–48.5)	Retrospective multi-center (electronic database)	China
Chen et al. [16]	203	M 108 F 95	54.0 (20–91)	Retrospective single center	China
Zhou et al. [17]	21	M 13 F 8	66.1 (SD 13.94)	Retrospective single center	China
Lo et al. [18]	10	M 3 F 7	54 (27–64)	Retrospective single center	China
Huang et al. [19]	41	M 30 F 11	49.0 (41.0–58.0)	Prospective multi-center (electronic database)	China
Zhang et al. [20]	645	M 328 F 317	46.65 (SD 13.82)	Retrospective multi-center (electronic database)	China
Chen et al. [21]	249	M 126 F 123	51.0 (36–64)	Retrospective single center	China
Feng et al. [22]	476	M 271 F 205	53.0 (40–64)	Retrospective multi-center	China
Chen et al. [23]	274	M 171 F 103	62.0 (44–70)	Retrospective single center	China
Zhang et al. [24]	140	M 71 F 69	57.0 (25–87)	Retrospective multi-center	China
Lian et al. [25]	788	M 407 F 381	41.15 (SD 11.38)	Retrospective multi-center (electronic database)	China
Cai et al. [26]	298	M 145 F 153	47.5 (33–61)	Retrospective single center	China
Wan et al. [27]	135	M 72 F 63	47.0 (36–55)	Retrospective single center	China
Cao et al. [28]	102	M 53 F 49	54.0 (37–67)	Retrospective single center	China
Wang et al. [29]	339	M 166 F 173	69.0 (65–76)	Retrospective single center	China
Xu et al. [30]	62	M 36 F 27	41.0 (32–52)	Retrospective single center	China
Zhou et al. [31]	191	M 119 F 72	56.0 (46–67)	Retrospective multi-center cohort study	China
Wu et al. [32]	201	M 128 F 73	51.0 (43–60)	Retrospective single center cohort study	China
Du et al. [33]	85	M 62 F 23	65.8	Retrospective multi-center	China
Wang et al. [34]	69	M 32 F 37	42.0 (35–62)	Retrospective single center	China
Guan et al. [35]	1099	M 640 F 459	47.0 (35–58)	Retrospective multi-center	China
Goyal et al. [36]	393	M 238 F 155	62.2 (49–74)	Retrospective multi-center	USA
Zhang et al. [37]	28	M 17 F 11	65.0 (56–70)	Retrospective single center	China
Chen et al. [38]	118	M 0 F 118	31.0 (28–34)	Retrospective single center	China
Wang et al. [39]	1012	M 524 F 488	50.0 (39–58)	Retrospective multi-center	China
Xia et al. [40]	10	M 6 F 4	56.5	Retrospective single center	China
Liang et al. [41]	1590	M 904 F 674	48.9 (SD 16.3)	Retrospective multi-center	China
Dai et al. [42]	234	M 136 F 98	44.6	Retrospective single center	China
Li et al. [43]	25	M 12 F 13	45.6	Retrospective single center	China
Chu et al. [44]	54	M 36 F 18	39	Retrospective single center	China
Qi et al. [45]	70	M 39 F 31	39.5	Retrospective multi-center	China
Godaert et al. [46]	17	M 8 F 9	86.5	Retrospective single center	France
Ye et al. [47]	5	M 2 F 3	30.0	Retrospective single center	China
Huang et al. [48]	22	M 6 F 16	22.0 (16.0–23.0)	Retrospective single center	China
Tian et al. [49]	262	M 127 F 135	47.5	Retrospective single center	China
Huang et al. [50]	34	M 14 F 20	56.2	Retrospective single center	China
Xia et al. [51]	20	M 13 F 7	1.5	Retrospective single center	China
Zhao et al. [52]	101	M 56 F 45	44.44	Retrospective multi-center	China
Xu et al. [53]	51	M 25 F 26	41.6	Retrospective single center	China
Li et al. [54]	548	M 279 F 269	60.0 (48–69)	Retrospective single center	China
Xu et al. [55]	90	M 39 F 51	50.0 (18–86)	Retrospective single center	China
Lei et al. [56]	119	M 77 F 42	49.0 (SD 13.6)	Retrospective multi-center	China
Pung et al. [57]	17	M 7 F 10	40.0	Retrospective single center	Singapore
Xu et al. [71]	50	M 29 F 21	42.3	Retrospective single center	China
Escalera-Antezana et al. [58]	12	M 6 F 6	36.5	Retrospective single center	Bolivia
Lechien et al. [59]	417	M 154 F 263	36.9 (SD 11.4)	Retrospective multi-center	Europe
Dong et al. [72]	11	M 5 F 6	40.3	Retrospective single center	China
Total: 54	12.046	M 6427 (54%) F 5597 (46%)	52.13

**Table 2:** Musculoskeletal symptoms

Study (year)	No. of patients	Fatigue (nr/%)	Arthralgia/Myalgia (nr/%)
Zheng et al. [37]	99	72 (73%)	12 (12%)
Lei et al. [36]	34	25 (73.5%)	11 (32.4%)
Mo et al. [60]	155	60 (73.2)	50 (61.0%)
Qian et al. [61]	91	40 (43.96%)	5 (5.49%)
Ma et al. [62]	37	4 (10.8%)	4 (10.8%)
Jin et al. [10]	651	119 (18.2%)	/
Zheng et al. [11]	161	64 (39.8%)	18 (11.2%)
Wang et al. [12]	80	28 (35%)	19 (23.75%)
Chen et al. [13]	203	16 (7.9%)	54 (26.6)
Zhou et al. [14]	21	5 (23.8%)	2 (9.5%)
Lo et al. [15]	10	/	3 (30%)
Huang et al. [16]	41	18 (44%)	/
Zhang et al. [17]	645	118 (18.3%)	71 (11%)
Chen et al. [18]	249	39 (15.7%)	/
Feng et al. [19]	476	/	59 (12.4%)
Chen et al. [20]	274	137 (50%)	60 (22%)
Zhang et al. [58]	140	105 (75%)	/
Lian et al. [47]	788	139 (17.6%)	91 (11.5%)
Cai et al. [21]	298	13 (4.3%)	/
Wan et al. [22]	135	/	44 (32.5%)
Cao et al. [9]	102	56 (54.9%)	35 (34.3)
Wang et al. [32]	339	135 (39.9%)	16 (4.7%)
Xu et al. [26]	62	/	32 (52%)
Zhou et al. [27]	191	44 (23%)	29 (15%)
Wu et al. [29]	201	65 (32.3%)	/
Du et al. [33]	85	50 (58.8%)	14 (16.5%)
Wang et al. [35]	69	29 (42%)	21 (30%)
Guan et al. [33]	1099	419 (38%)	164 (15%)
Goyal et al. [46]	393	/	94 (24%)
Zhang et al. [24]	28	18 (64%)	4 (14%)
Chen et al. [34]	118	19 (16%)	/
Wang et al. [41]	1012	/	170 (17%)
Xia et al. [38]	10	3 (30%)	/
Liang et al. [39]	1590	680 (43%)	278 (17%)
Dai et al. [40]	234	31 (13%)	21 (9%)
Li et al. [41]	25	17 (68%)	/
Chu et al. [42]	54	9 (17%)	3 (6%)
Qi et al. [43]	70	/	12 (17%)
Godaert et al. [7]	17	10 (59%)	/
Ye et al. [28]	5	5 (100%)	/
Huang et al. [44]	22	5 (23%)	4 (18%)
Tian et al. [45]	262	69 (26%)	/
Huang et al. [8]	34	/	22 (65%)
Xia et al. [48]	20	1 (5%)	/
Zhao et al. [49]	101	/	17 (17%)
Xu et al. [25]	51	2 (4%)	8 (16%)
Li et al. [51]	548	258 (47%)	111 (20%)
Xu et al. [52]	90	19 (21%)	25 (28%)
Lei et al. [54]	119	/	18 (15%)
Pung et al. [6]	17	/	5 (29%)
Xu et al. [55]	50	8 (16%)	8 (16%)
Escalera-Antezana et al. [59]	12	/	5 (42%)
Lechien et al. [57]	417	129 (31%)	246 (59%)
Dong et al. [56]	11	2 (18%)	1 (9%)
Total: 54	Tot: 12,046	3085 (25.6%)	1866 (15.5%)

Data on 12,046 patients (54% male and 46% females) were available. The number of patients in the selected studies ranged from 5 to 1590 patients (223 ± 312 patients). The sex ratio (male to female) was 1:15, and the overall average of patients was 52.13 years. The majority of the studies arose from China, mainly from Wuhan; one was from Singapore [57], two from Europe [46, 59], one from the USA [36], and one from Bolivia [58]. Musculoskeletal symptoms were present from the earliest stage of the viral illness and were reported in patients necessitating intensive care in the end stage of the condition. The total prevalence of fatigue symptom was 25.6% (*R*^2^ =0.56; *p* value = 0.004), while the prevalence of arthralgia and/or myalgia was 15.5% (*R*^2^ = 0.66; *p* value = 0.001; Fig. 1).

**Fig. 1:**
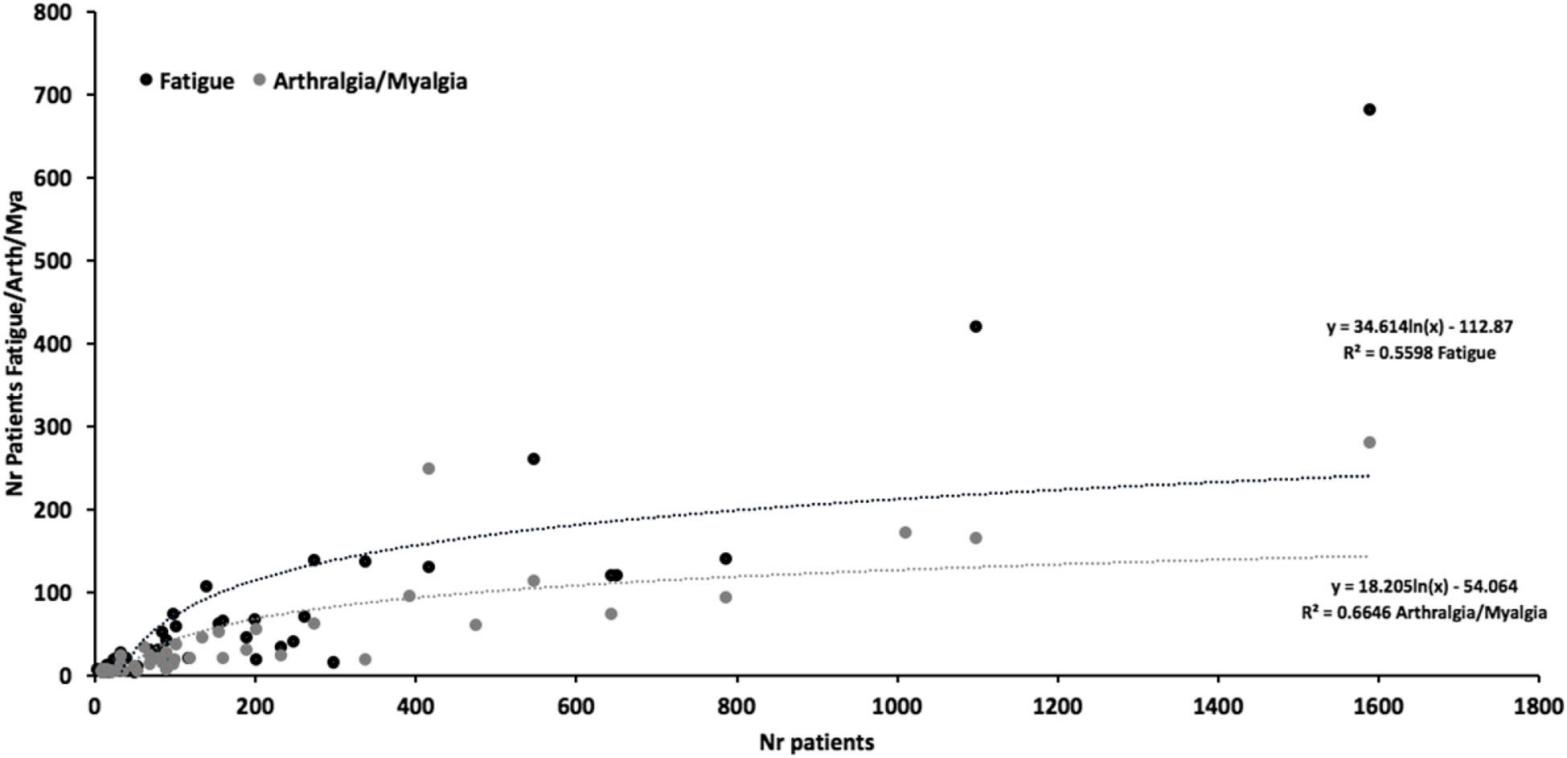
Relationships of fatigue and arthralgia/myalgia on all patients

Eight studies reported a prevalence higher than 50% of patients with fatigue [8, 9, 24, 25, 28, 37, 46, 47], while three studies reported higher values for arthralgia/myalgia symptoms [50, 53, 59]. The prevalence of musculoskeletal symptoms in studies from Europe reached high values [46, 59]; Lechien et al., for example, reported on 417 COVID-19 patients from 12 European hospitals and found myalgia in 246 (59%) and arthralgia in 129 (31%) of these patients [59].

Clinical presentation of COVID-19 ranges from absence of symptoms to severe pneumonia. Fever, dry cough and fatigue are common symptoms, as indeed are myalgia and arthralgia [6, 53]. Most of the articles are retrospective single center studies: data were collected in a non-homogeneous way, especially regarding comorbidities, lifestyle habits, and severity of the illness. Based on our work, we cannot state, for example, whether children and younger patients less commonly present musculoskeletal symptoms at onset [63]. Most studies originate from China, which is not surprising, and it is not clear whether the prevalence of musculoskeletal symptoms at onset is influenced by socio-geographical factors [64]. The most common symptoms in patients with mild to moderate clinical presentation of the condition are fever, fatigue, and dry cough, followed by other symptoms including headache, nasal congestion, sore throat, myalgia, and arthralgia [65, 66].

The evidence on the central role of inflammation during COVID-19 infection underlines the need to block this inflammatory cascade [30, 60–62, 67–70]. The presence of musculoskeletal symptoms is worrying: there is a high rate of use, especially in the middle age and elderly population, of NSAIDs. The fact that patients therefore report musculoskeletal symptoms is even more worrying because it may imply that the inflammatory reactions overcome the anti-inflammatory effect of such drugs.

Clinical features have to be analyzed deeply, especially considering the new evidences on COVID-19. Musculoskeletal symptoms should be married with laboratory findings, such as inflammatory and infection-related parameters (Interleukin-6, Procalcitonin, C-reactive protein). Understandably, the involvement of the musculoskeletal system has not been deeply investigated during this pandemic, but synovial and muscle biopsy, and joint fluid analysis, for example, should clarify how extensive the attack of the virus on the whole of the human body is. Until now, no report has been published on the presence of COVID-19 in the skeletal muscles, joint, or bones. The musculoskeletal symptoms are only anecdotally attributed to indirect effects, mainly arising from inflammatory and/or immune response, but other mechanisms can be hypothesized, such as direct damage by the virus on the endothelium or peripheral nerves. These findings could help to plan specific rehabilitation protocols in COVID-19 patients.

As a new infectious disease, it is particularly important to underline the clinical features of COVID-19, especially in the early stage of the illness, to help clinicians to individuate and isolate patients earlier, and then minimize its diffusion. From the onset of the symptoms and to the most severe stages of COVID-19 disease, musculoskeletal symptoms, including myalgia, arthralgia, and fatigue, are a nearly constant presence. It is still unclear how the effects of COVID-19 on the musculoskeletal system are mediated.

## References

[1] Park M, Cook AR, Lim JT, et al. A systematic review of COVID-19 epidemiology based on current evidence. J Clin Med. 2020;**9**. 10.3390/jcm9040967. 10.3390/jcm9040967 PMC7231098 32244365

[2] Fu L, Wang B, Yuan T, et al. Clinical characteristics of coronavirus disease 2019 (COVID-19) in China: a systematic review and meta-analysis. J Infect Published Online First: 10 April 2020. doi:10.1016/j.jinf.2020.03.041. 10.1016/j.jinf.2020.03.041 PMC7151416 32283155

[3] Zhu Z, Cai T, Fan L, et al. Clinical value of immune-inflammatory parameters to assess the severity of coronavirus disease 2019. Int J Infect Dis Published Online First: 22 April 2020. doi:10.1016/j.ijid.2020.04.041. 10.1016/j.ijid.2020.04.041 PMC7195003 32334118

[4] Kaiser L, Fritz RS, Straus SE (2001). Symptom pathogenesis during acute influenza: interleukin-6 and other cytokine responses. J Med Virol.

[5] Misra DP, Agarwal V, Gasparyan AY, et al. Rheumatologists’ perspective on coronavirus disease 19 (COVID-19) and potential therapeutic targets. Clin Rheumatol Published Online First: 10 April 2020. doi:10.1007/s10067-020-05073-9. 10.1007/s10067-020-05073-9 PMC7145936 32277367

[6] Pung R, Chiew CJ, Young BE (2020). Investigation of three clusters of COVID-19 in Singapore: implications for surveillance and response measures. Lancet.

[7] Godaert L, Proye E, Demoustier-Tampere D, et al. Clinical characteristics of older patients: the experience of a geriatric short-stay unit dedicated to patients with COVID-19 in France. *J Infect* Published Online First: 17 April 2020. doi:10.1016/j.jinf.2020.04.009. 10.1016/j.jinf.2020.04.009 PMC7162788 32305489

[8] Huang Y, Tu M, Wang S, et al. Clinical characteristics of laboratory confirmed positive cases of SARS-CoV-2 infection in Wuhan, China: A retrospective single center analysis. Travel Med Infect Dis 2020;101606. doi:10.1016/j.tmaid.2020.101606. 10.1016/j.tmaid.2020.101606 PMC7102650 32114074

[9] Cao J, Tu W-J, Cheng W, et al. Clinical features and short-term outcomes of 102 patients with Corona Virus Disease 2019 in Wuhan, China. Clin Infect Dis Published Online First: 2 April 2020. doi:10.1093/cid/ciaa243. 10.1093/cid/ciaa243 PMC7184479 32239127

[10] Jin X, Lian J-S, Hu J-H, *et al.* Epidemiological, clinical and virological characteristics of 74 cases of coronavirus-infected disease 2019 (COVID-19) with gastrointestinal symptoms. *Gut* Published Online First: 24 March 2020. doi:10.1136/gutjnl-2020-320926. 10.1136/gutjnl-2020-320926 PMC7133387 32213556

[11] Zheng F, Tang W, Li H (2020). Clinical characteristics of 161 cases of corona virus disease 2019 (COVID-19) in Changsha. Eur Rev Med Pharmacol Sci.

[12] Wang X, Liu W, Zhao J, et al. Clinical characteristics of 80 hospitalized frontline medical workers infected with COVID-19 in Wuhan, China. J Hosp Infect Published Online First: 14 April 2020. doi:10.1016/j.jhin.2020.04.019. 10.1016/j.jhin.2020.04.019 PMC7194674 32302722

[13] Chen T, Dai Z, Mo P, et al. Clinical characteristics and outcomes of older patients with coronavirus disease 2019 (COVID-19) in Wuhan, China (2019): a single-centered, retrospective study. J Gerontol A Biol Sci Med Sci Published Online First: 11 April 2020. doi:10.1093/gerona/glaa089. 10.1093/gerona/glaa089 PMC7184388 32279081

[14] Zhou Y, Han T, Chen J, et al. Clinical and autoimmune characteristics of severe and critical cases with COVID-19. Clin Transl Sci Published Online First: 21 April 2020. doi:10.1111/cts.12805. 10.1111/cts.12805 PMC7264560 32315487

[15] Lo IL, Lio CF, Cheong HH (2020). Evaluation of SARS-CoV-2 RNA shedding in clinical specimens and clinical characteristics of 10 patients with COVID-19 in Macau. Int J Biol Sci.

[16] Huang C, Wang Y, Li X (2020). Clinical features of patients infected with 2019 novel coronavirus in Wuhan, China. Lancet.

[17] Zhang X, Cai H, Hu J (2020). Epidemiological, clinical characteristics of cases of SARS-CoV-2 infection with abnormal imaging findings. Int J Infect Dis.

[18] Chen J, Qi T, Liu L (2020). Clinical progression of patients with COVID-19 in Shanghai, China. J Infect.

[19] Feng Y, Ling Y, Bai T, et al. COVID-19 with different severity: a multi-center study of clinical features. Am J Respir Crit Care Med Published Online First: 10 April y. doi:10.1164/rccm.202002-0445OC.

[20] Chen T, Wu D, Chen H (2020). Clinical characteristics of 113 deceased patients with coronavirus disease 2019: retrospective study. BMJ.

[21] Cai Q, Huang D, Ou P, et al. COVID-19 in a designated infectious diseases hospital outside Hubei Province, China. Allergy Published Online First: 2 April 2020. doi:10.1111/all.14309. 10.1111/all.14309 32239761

[22] Wan S, Xiang Y, Fang W, et al. Clinical features and treatment of COVID-19 patients in northeast Chongqing. J Med Virol Published Online First: 21 March 2020. doi:10.1002/jmv.25783. 10.1002/jmv.25783 PMC7228368 32198776

[23] Wang L, He W, Yu X, et al. Coronavirus disease 2019 in elderly patients: characteristics and prognostic factors based on 4-week follow-up. J Infect Published Online First: 30 March y. doi:10.1016/j.jinf.2020.03.019. 10.1016/j.jinf.2020.03.019 PMC7118526 32240670

[24] Zhang L, Zhu F, Xie L, et al. Clinical characteristics of COVID-19-infected cancer patients: a retrospective case study in three hospitals within Wuhan, China. Ann Oncol Published Online First: 26 March 2020. doi:10.1016/j.annonc.2020.03.296. 10.1016/j.annonc.2020.03.296 PMC7270947 32224151

[25] Xu T, Chen C, Zhu Z (2020). Clinical features and dynamics of viral load in imported and non-imported patients with COVID-19. Int J Infect Dis.

[26] Xu X-W, Wu X-X, Jiang X-G (2020). Clinical findings in a group of patients infected with the 2019 novel coronavirus (SARS-Cov-2) outside of Wuhan, China: retrospective case series. BMJ.

[27] Zhou F, Yu T, Du R (2020). Clinical course and risk factors for mortality of adult inpatients with COVID-19 in Wuhan, China: a retrospective cohort study. Lancet.

[28] Ye F, Xu S, Rong Z (2020). Delivery of infection from asymptomatic carriers of COVID-19 in a familial cluster. Int J Infect Dis.

[29] Wu C, Chen X, Cai Y, et al. Risk factors associated with acute respiratory distress syndrome and death in patients with Coronavirus Disease 2019 pneumonia in Wuhan, China. JAMA Intern Med Published Online First: 13 March 2020. doi:10.1001/jamainternmed.2020.0994. 10.1001/jamainternmed.2020.0994 PMC7070509 32167524

[30] Lippi G, Plebani M (2020). Procalcitonin in patients with severe coronavirus disease 2019 (COVID-19): a meta-analysis. Clin Chim Acta.

[31] Du Y, Tu L, Zhu P, et al. Clinical features of 85 fatal cases of COVID-19 from Wuhan: a retrospective observational study. Am J Respir Crit Care Med Published Online First: 3 April 2020. doi:10.1164/rccm.202003-0543OC. 10.1164/rccm.202003-0543OC PMC7258652 32242738

[32] Wang Z, Yang B, Li Q, et al. Clinical features of 69 cases with Coronavirus Disease 2019 in Wuhan, China. Clin Infect Dis Published Online First: 16 March 2020. doi:10.1093/cid/ciaa272. 10.1093/cid/ciaa272 PMC7184452 32176772

[33] Guan W, Ni Z, Hu Y, et al. Clinical characteristics of Coronavirus Disease 2019 in China. New England Journal of Medicine 2020;**0**:null. doi:10.1056/NEJMoa2002032. 10.1056/NEJMoa2002032 PMC7092819 32109013

[34] Chen L, Li Q, Zheng D, et al. Clinical characteristics of pregnant women with Covid-19 in Wuhan, China. N Engl J Med Published Online First: 17 April 2020. doi:10.1056/NEJMc2009226. 10.1056/NEJMc2009226 PMC7182016 32302077

[35] Wang X, Fang J, Zhu Y, et al. Clinical characteristics of non-critically ill patients with novel coronavirus infection (COVID-19) in a Fangcang Hospital. Clin Microbiol Infect Published Online First: 3 April 2020. doi:10.1016/j.cmi.2020.03.032. 10.1016/j.cmi.2020.03.032 PMC7195539 32251842

[36] Lei S, Jiang F, Su W, et al. Clinical characteristics and outcomes of patients undergoing surgeries during the incubation period of COVID-19 infection. EClinicalMedicine 2020;:100331. doi:10.1016/j.eclinm.2020.100331. 10.1016/j.eclinm.2020.100331 PMC7128617 32292899

[37] Zheng Y, Xu H, Yang M (2020). Epidemiological characteristics and clinical features of 32 critical and 67 noncritical cases of COVID-19 in Chengdu. J Clin Virol.

[38] Xia X-Y, Wu J, Liu H-L (2020). Epidemiological and initial clinical characteristics of patients with family aggregation of COVID-19. J Clin Virol.

[39] Liang W-H, Guan W-J, Li C-C, et al. Clinical characteristics and outcomes of hospitalised patients with COVID-19 treated in Hubei (epicenter) and outside Hubei (non-epicenter): a nationwide analysis of China. Eur Respir J Published Online First: 8 April 2020. doi:10.1183/13993003.00562-2020. 10.1183/13993003.00562-2020 PMC7144336 32269086

[40] Dai H, Zhang X, Xia J (2020). High-resolution chest CT features and clinical characteristics of patients infected with COVID-19 in Jiangsu, China. Int J Infect Dis.

[41] Li X, Wang L, Yan S (2020). Clinical characteristics of 25 death cases with COVID-19: a retrospective review of medical records in a single medical center, Wuhan, China. Int J Infect Dis.

[42] Chu J, Yang N, Wei Y, et al. Clinical characteristics of 54 medical staff with COVID-19: a retrospective study in a single center in Wuhan, China. J Med Virol Published Online First: 29 March 2020. doi:10.1002/jmv.25793. 10.1002/jmv.25793 PMC7228263 32222986

[43] Qi X, Liu C, Jiang Z, et al. Multicenter analysis of clinical characteristics and outcome of COVID-19 patients with liver injury. J Hepatol Published Online First: 16 April 2020. doi:10.1016/j.jhep.2020.04.010.

[44] Huang L, Zhang X, Zhang X, et al. Rapid asymptomatic transmission of COVID-19 during the incubation period demonstrating strong infectivity in a cluster of youngsters aged 16-23 years outside Wuhan and characteristics of young patients with COVID-19: a prospective contact-tracing study. J Infect Published Online First: 10 April 2020. doi:10.1016/j.jinf.2020.03.006. 10.1016/j.jinf.2020.03.006 PMC7194554 32283156

[45] Tian S, Hu N, Lou J (2020). Characteristics of COVID-19 infection in Beijing. J Infect.

[46] Goyal P, Choi JJ, Pinheiro LC, et al. Clinical characteristics of Covid-19 in New York City. N Engl J Med Published Online First: 17 April 2020. doi:10.1056/NEJMc2010419. 10.1056/NEJMc2010419 PMC7182018 32302078

[47] Lian J, Jin X, Hao S, et al. Analysis of epidemiological and clinical features in older patients with Corona Virus Disease 2019 (COVID-19) out of Wuhan. Clin Infect Dis Published Online First: 25 March 2020. doi:10.1093/cid/ciaa242. 10.1093/cid/ciaa242 PMC7184356 32211844

[48] Xia W, Shao J, Guo Y (2020). Clinical and CT features in pediatric patients with COVID-19 infection: different points from adults. Pediatr Pulmonol.

[49] Zhao W, Zhong Z, Xie X (2020). Relation between chest CT findings and clinical conditions of coronavirus disease (COVID-19) Pneumonia: A Multicenter Study. AJR Am J Roentgenol.

[50] Vetter P, Vu DL, L’Huillier AG, et al. Clinical features of covid-19. BMJ. 2020;**369**. 10.1136/bmj.m1470. 10.1136/bmj.m1470 32303495

[51] Li X, Xu S, Yu M, et al. Risk factors for severity and mortality in adult COVID-19 inpatients in Wuhan. J Allergy Clin Immunol Published Online First: 12 April 2020. doi:10.1016/j.jaci.2020.04.006. 10.1016/j.jaci.2020.04.006 PMC7152876 32294485

[52] Xu X, Yu C, Qu J (2020). Imaging and clinical features of patients with 2019 novel coronavirus SARS-CoV-2. Eur J Nucl Med Mol Imaging.

[53] Cruz AT, Zeichner SL. COVID-19 in children: initial characterization of the pediatric disease. Pediatrics Published Online First: 16 March 2020. doi:10.1542/peds.2020-0834. 10.1542/peds.2020-0834 32179659

[54] Lei Z, Cao H, Jie Y, et al. A cross-sectional comparison of epidemiological and clinical features of patients with coronavirus disease (COVID-19) in Wuhan and outside Wuhan, China. Travel Med Infect Dis 2020;:101664. doi:10.1016/j.tmaid.2020.101664. 10.1016/j.tmaid.2020.101664 PMC7194579 32278758

[55] Xu Y-H, Dong J-H, An W-M (2020). Clinical and computed tomographic imaging features of novel coronavirus pneumonia caused by SARS-CoV-2. J Infect.

[56] Dong X, Cao Y-Y, Lu X-X, et al. Eleven faces of coronavirus disease 2019. Allergy Published Online First: 20 March 2020. doi:10.1111/all.14289. 10.1111/all.14289 PMC7228397 32196678

[57] Lechien JR, Chiesa-Estomba CM, De Siati DR, et al. Olfactory and gustatory dysfunctions as a clinical presentation of mild-to-moderate forms of the coronavirus disease (COVID-19): a multicenter European study. Eur Arch Otorhinolaryngol Published Online First: 6 April 2020. doi:10.1007/s00405-020-05965-1. 10.1007/s00405-020-05965-1 PMC7134551 32253535

[58] Zhang J-J, Dong X, Cao Y-Y, et al. Clinical characteristics of 140 patients infected with SARS-CoV-2 in Wuhan, China. Allergy Published Online First: 19 February 2020. doi:10.1111/all.14238. 10.1111/all.14238 32077115

[59] Escalera-Antezana JP, Lizon-Ferrufino NF, Maldonado-Alanoca A, et al. Clinical features of the first cases and a cluster of Coronavirus Disease 2019 (COVID-19) in Bolivia imported from Italy and Spain. Travel Med Infect Dis 2020;:101653. doi:10.1016/j.tmaid.2020.101653. 10.1016/j.tmaid.2020.101653 PMC7129170 32247926

[60] Mo P, Xing Y, Xiao Y, et al. Clinical characteristics of refractory COVID-19 pneumonia in Wuhan, China. Clin Infect Dis Published Online First: 16 March 2020. doi:10.1093/cid/ciaa270.

[61] Qian G-Q, Yang N-B, Ding F, et al. Epidemiologic and clinical characteristics of 91 hospitalized patients with COVID-19 in Zhejiang, China: a retrospective, multi-centre case series. QJM Published Online First: 17 March 2020. doi:10.1093/qjmed/hcaa089. 10.1093/qjmed/hcaa089 PMC7184349 32181807

[62] Ma J, Yin J, Qian Y, et al. Clinical characteristics and prognosis in cancer patients with COVID-19: a single center’s retrospective study. J Infect Published Online First: 14 April 2020. doi:10.1016/j.jinf.2020.04.006. 10.1016/j.jinf.2020.04.006 PMC7194544 32298677

[63] Wang C, Pan R, Wan X, et al. Immediate psychological responses and associated factors during the initial stage of the 2019 Coronavirus Disease (COVID-19) epidemic among the general population in China. Int J Environ Res Public Health 2020;**17**. doi:10.3390/ijerph17051729. 10.3390/ijerph17051729 PMC7084952 32155789

[64] Chen N, Zhou M, Dong X (2020). Epidemiological and clinical characteristics of 99 cases of 2019 novel coronavirus pneumonia in Wuhan, China: a descriptive study. Lancet.

[65] Yang X, Yu Y, Xu J, et al. Clinical course and outcomes of critically ill patients with SARS-CoV-2 pneumonia in Wuhan, China: a single-centered, retrospective, observational study. Lancet Respir Med Published Online First: 24 February 2020. 10.1016/S2213-2600(20)30079-5. 10.1016/S2213-2600(20)30079-5 PMC7102538 32105632

[66] Zhou W, Liu Y, Tian D (2020). Potential benefits of precise corticosteroids therapy for severe 2019-nCoV pneumonia. Sig Transduct Target Ther.

[67] Fang X, Mei Q, Yang T, et al. Low-dose corticosteroid therapy does not delay viral clearance in patients with COVID-19. J Infect Published Online First: 11 April 2020. doi:10.1016/j.jinf.2020.03.039. 10.1016/j.jinf.2020.03.039 PMC7151466 32283153

[68] Gerlach H (2016). Agents to reduce cytokine storm. F1000Res.

[69] Mehta P, McAuley DF, Brown M (2020). COVID-19: consider cytokine storm syndromes and immunosuppression. Lancet.

[70] Ritchie AI, Singanayagam A (2020). Immunosuppression for hyperinflammation in COVID-19: a double-edged sword?. Lancet.

[71] Xu Y-H, Dong J-H, An W-M, Lv XY, Yin XP, Zhang JZ, Dong L, Ma X, Zhang HJ, Gao BL. Clinical and computed tomographic imaging features of novel coronavirus pneumonia caused by SARS-CoV-2. J Infect. 2020;80:394–400. 10.1016/j.jinf.2020.02.017. 10.1016/j.jinf.2020.02.017 PMC7102535 32109443

[72] Dong X, Cao YY, Lu XX, Zhang JJ, Du H, Yan YQ, Akdis CA, Gao YD. Eleven faces of coronavirus disease 2019. Allergy. 2020. 10.1111/all.14289. [Epub ahead of print]. 10.1111/all.14289 PMC7228397 32196678

